# Aptamer–Protein Structures Guide In Silico and Experimental Discovery of Aptamer–Short Peptide Recognition Complexes or Aptamer–Amino Acid Cluster Complexes

**DOI:** 10.1021/acs.jpcb.2c05624

**Published:** 2022-10-31

**Authors:** Michael Fadeev, Michael P. O’Hagan, Yonatan Biniuri, Itamar Willner

**Affiliations:** The Institute of Chemistry, The Centre of Nanoscience and Nanotechnology, The Hebrew University of Jerusalem, Jerusalem91904, Israel

## Abstract

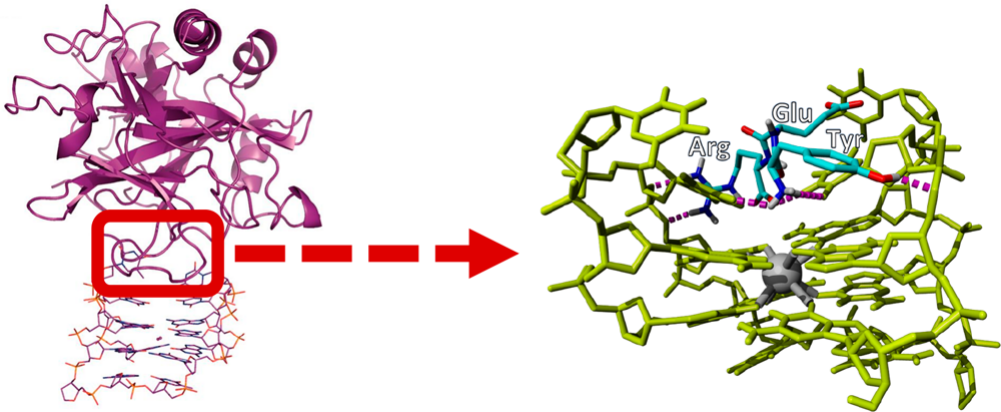

A method to computationally and experimentally identify aptamers against short peptides or amino acid clusters is introduced. The method involves the selection of a well-defined protein aptamer complex and the extraction of the peptide sequence participating in the binding of the protein to the aptamer. The subsequent fragmentation of the peptide sequence into short peptides and the in silico docking-guided identification of affinity complexes between the miniaturized peptides and the antiprotein aptamer, followed by experimental validation of the binding features of the short peptides with the antiprotein aptamers, leads to the identification of new short peptide-aptamer complexes. This is exemplified with the identification of the pentapeptide RYERN as the scaffold that binds thrombin to the DNA thrombin aptamer (DNA TA). In silico docking studies followed by microscale thermophoresis (MST) experiments demonstrate that the miniaturized tripeptides RYE, YER, and ERN reveal selective binding affinities toward the DNA TA. In addition, docking and MST experiments show that the ribonucleotide-translated RNA TA shows related binding affinities of YER to the DNA TA. Most importantly, we demonstrate that the separated amino acids Y/E/R assemble as a three amino acid cluster on the DNA TA and RNA TA aptamers in spatial configurations similar to the tripeptide YER on the respective aptamers. The clustering phenomenon is selective for the YER tripeptide system. The method to identify binding affinities of miniaturized peptides to known antiprotein aptamers and the specific clustering of single amino acids on the aptamers is further demonstrated by in silico and experimental identification of the binding of the tripeptide RET and the selective clustering of the separated amino acids R/E/T onto a derivative of the AS1411 aptamer against the nucleolin receptor protein.

## Introduction

Aptamers are single-stranded DNA or RNA oligonucleotide biopolymers revealing base-dictated three-dimensional binding interactions toward low-molecular-weight substrates, macromolecules, and even cells.^1,2^ The base-controlled folding within the aptamer-ligand complexes yields selective and specific supramolecular complexes. The in vitro eliciting of aptamers is based on the selection and amplification of the sequence-specific binding strands from a diversified library of nucleic acids using a systematic evolution by exponential ligand enrichment (SELEX) protocol.^3−5^ Different applications of aptamers were reported including their use for sensing (aptasensors),^6−11^ imaging,^12^ and therapeutic applications, for example, the use of aptamers as gating units of drug carriers for controlled release by biomarkers,^13,14^ targeting of drugs to cell-specific targets,^15,16^ inhibition of harmful proteins,^17,18^ or the generation of active drugs such as Zn(II) protoporphyrin bound to the G-quadruplex VEGF aptamer/VEGF complex for photodynamic therapy.^19^ The linking of aptamer strands to DNAzymes,^20,21^ homogeneous catalysts,^22,23^ and heterogeneous catalytic nanoparticles^24^ generated supramolecular structures mimicking native enzymes (“nucleoapzymes” and “aptananozymes”). Also, conjugation of aptamers to photosensitizer units allowed the assembly of supramolecular artificial photosynthetic systems.^25,26^ Substantial efforts were directed to develop means to improve the binding affinities of aptamers and to control their binding affinities by chemical functionalities. These included the mutation of the aptamer bases in the aptamer binding domain,^27^ the assembly of bivalent aptamer constructs,^28^ the incorporation of artificial nucleobases into the aptamer sequences,^29,30^ and tethering of molecular chemical constituents to the biopolymer.^31^ Also, in silico aptamer design and modeling methods were suggested to construct aptamers with improved binding affinities.^32^ Control over the binding affinities of aptamers by stimuli-responsive chemical functionalities tethered to the aptamer backbones was demonstrated. For example, tethering of methylene blue to the adenosine triphosphate (ATP) aptamer yielded redox-switchable aptamers revealing ON/OFF binding affinities in the presence of reducing or oxidizing agents or under electrochemical control.^33^ Moreover, reversible photochemically controlled binding of the thrombin aptamer to thrombin was demonstrated by chemical modification of the aptamer with photoisomerizable *cis–trans* azobenzene intercalator units.^34^ In addition, aptamer binding affinities were blocked by photocleavable *ortho*-nitrobenzylphosphate locks that were photochemically uncaged to activate the binding functions of aptamers.^35^

Here we wish to report the identification of short peptides revealing binding affinities toward DNA (or RNA) aptamers previously elicited against macromolecular protein scaffolds. The selection of potential short peptides that may bind to the aptamers is initially guided by the X-ray resolved three-dimensional structure of a previously reported protein-aptamer complex and, subsequently, by identification of a protein exhibiting similar structural features to the first example, for which a structurally related aptamer was also elicited. We apply this method to identify a series of small peptides that bind to the DNA thrombin aptamer and a modified form of the AS1411 nucleolin-binding aptamer. Computational docking simulations predict the binding model of the peptides to the aptamers, and these are validated by experiments. For some of the peptides, we find that the single amino acids that comprise the peptides cluster as supramolecular nanostructures at the peptide binding sites. The binding of the peptides and amino acid clusters to the DNA/RNA sequences are selective and specific. The significance of this study is reflected by the introduction of a versatile means to identify stable affinity complexes between protein binding aptamers and miniaturized peptide ligands. This paves the way to further modify the peptide ligands with chemical functionalities for diverse applications. Beyond the significance of the study in advancing the area of aptamer-based nanotechnology, the identification of specific RNA/peptide or RNA/amino acid interactions might shed light on analogous interactions within the RNA/peptide world under prebiotic conditions and evolution of life.^36−38^

## Experimental Section

### Oligonucleotides and Peptides

Fluorescein amidite (FAM)-labeled oligonucleotides for MST analysis were purchased from Integrated DNA Technologies (IDT). The sequences used were DNA TA: 5′-FAM-d[GGTTGGTGTGGTTGG]; RNA TA: 5′-FAM-r[GGUUGGUGUGGUUGG]; 2N3M: 5′-FAM-d[TGGTGGTGGTTGTTGTGGTGGTGGTGGT]. Unlabeled DNA TA for NMR analysis was purchased from Sigma-Aldrich. Peptides (>95% purity) were obtained from Syntezza Bioscience. Amino acids (>99% purity) were purchased from Sigma-Aldrich. Stock solutions of oligonucleotides (100–200 μM), peptides (20 mM), and amino acids (20 mM) were prepared in triple-distilled H_2_O.

### Docking Studies

Docking studies were performed in YASARA software (http://www.yasara.org/).^39^ Structures of the PDB ID: 4DIH (DNA TA)^40,41^ and PDB ID: 2N3M(42,43) G-quadruplexes were obtained from the RCSB PDB (https://www.rcsb.org/).^44^ The structures of amino acids and peptides were constructed in YASARA Structure (ver. 21.12.19). Prior to docking, the receptor energy was minimized in YASARA Structure using the built-in macro ‘em_run’. Docking simulations were performed in YASARA using the built-in macro ‘dock_run’ with a flexible ligand. For the DNA TA, 100 docking runs were performed using the AutoDockLGA algorithm with the AMBER03 force field.^45^ For 2N3M, 100 docking runs were performed using the AutoDockVINA algorithm.^46^ The simulated structures were clustered with a 5 Å RMSD cutoff, and the dissociation constant of the energy minimized cluster was obtained from the simulation report. The calculated binding interactions of the energy minimized cluster were visualized in the YASARA software, and graphics were produced using POVRay (www.povray.org).

### Microscale Thermophoresis

A 1:1 serial dilution of the appropriate peptide or amino acid combination (2 × final concentration) was prepared in 25 mM phosphate-buffered saline (PBS), pH 7.05, that contained 100 mM potassium chloride (DNA TA and RNA TA) or 25 mM potassium phosphate buffer, pH 7.05, that contained 100 mM potassium chloride (2N3M). To 10 μL of each of the serial dilutions, 10 μL of a 400 nM (2 × final concentration) stock solution of labeled oligonucleotide in appropriate buffer was added to yield a final receptor concentration of 200 nM. The final samples were incubated for 10 min at room temperature. Subsequently, each sample was loaded into a NanoTemper KM-022 capillary tube and mounted on a NanoTemper Monolith Nt.115 capillary tray. Samples were irradiated using the “Blue” excitation wavelength setting with the light-emitting diode (LED) power set to obtain a fluorescence intensity of ca. 1000 units. The temperature was 25 °C (DNA) or 40 °C (RNA). For each MST measurement, each capillary was subjected to an IR laser at 10–20% power to record the resulting thermophoretic curves. The experimental MST profile consisted of a cold region of 5 s, followed by a hot (IR heated) region of 30 s, followed by a 5 s cold region to observe the regeneration of the fluorescence signal characteristic of the cold region value. Experiments were performed in duplicate. Binding curves were obtained using Origin 2022. Dissociation constants were extracted from the resulting binding curves using a Hill fitting.

### NMR Studies

^1^H one-dimensional (1D) and two-dimensional (2D) nuclear Overhauser spectroscopy (NOESY) spectra of DNA TA were recorded on a Bruker Avance 500 MHz spectrometer at 298 K. Water suppression was performed using the Watergate W5 sequence.^47^ The oligonucleotide (500 μM) was dissolved in 25 mM potassium phosphate buffer, pH 7.4, containing 100 mM KCl and 10% D_2_O. Following acquisition of the oligonucleotide ^1^H or 2D NOESY spectra,^48^ 30 μL of 20 mM YER stock solution was added to the sample to afford a final concentration of 1 mM YER (2 equiv) before the spectra of the DNA TA/YER complex were recorded. The nuclear Overhauser effect (NOE) mixing time was 150 ms. Spectra were processed in Topspin (Bruker), and resonances were assigned from previously reported data.^49^

## Results and Discussion

It is well-established that sequence-specific aptamers against proteins or low-molecular-weight substrates can be elicited by the SELEX procedure. The resulting affinity complexes between the ligands and the aptamer reveal specific interactions between the ligands and the constituent bases of the biopolymer. We reasoned that, upon taking a protein that exhibits well-resolved interactions between the aptamer nucleotide bases and the protein amino acids, one could identify well-defined miniaturized peptides that potentially bind to the aptamer sequence of the parent protein. By computational docking experiments, the formation of potential affinity complexes between the miniaturized peptides and the aptamer may be probed, and the putative peptide–aptamer complexes may be subsequently validated by binding experiments. As a proof-of-concept, we selected the antithrombin G-quadruplex DNA aptamer (DNA TA) as an aptamer scaffold that could potentially bind miniaturized peptides. The three-dimensional structure of the DNA TA/thrombin complex was extensively explored by X-ray methods, and the full structure of the complex is available in the RCSB PDB (https://www.rcsb.org/),^44^ PDB ID: 4DIH.^40,41^Figure S1 depicts the reported structural features between the DNA TA and thrombin. Based on this structure we identified the pentapeptide RYERN in the protruding loop region of the protein as the binding hotspot that is recognized by the DNA TA aptamer. This peptide scaffold was then fragmented to the three tripeptides RYE, YER, and ERN as potential miniaturized peptides that may bind the DNA TA.

Docking simulations find growing interest as computational means to evaluate the structures and binding affinities of aptamer–ligand complexes.^50^ Among the available simulation programs, the YASARA software provides a useful toolbox to systematically model the structural features and energy-minimized configurations of biomaterial complexes. Indeed, YASARA was successfully applied to evaluate the energy-minimized structures of a series of ligand–aptamer complexes such as argininamide and its derivatives^51^ to the argininamide aptamer and the binding of ATP to the ATP aptamer and its mutants.^27^ Following the evaluation of the energy-minimized structures of the aptamer-ligand complexes, the software returns the simulated dissociation constants (*K*_d_) of the structures. General features of the YASARA software include built-in AutoDock with the Lamarkian Genetic Algorithm and AMBER force fields^45^ and the AutoDock VINA docking algorithm^46^ previously employed in docking studies of nucleic acids.^52^ Accordingly, the energy-minimized structures of the miniaturized peptides to the DNA or RNA TAs and the resulting simulated *K*_d_ values of the complex were probed using the YASARA docking macro, in which 100 docking simulations on each peptide/ligand combination were performed. The lowest-energy binding poses were examined for their binding features to the DNA and RNA TAs.

**Figure 1:**
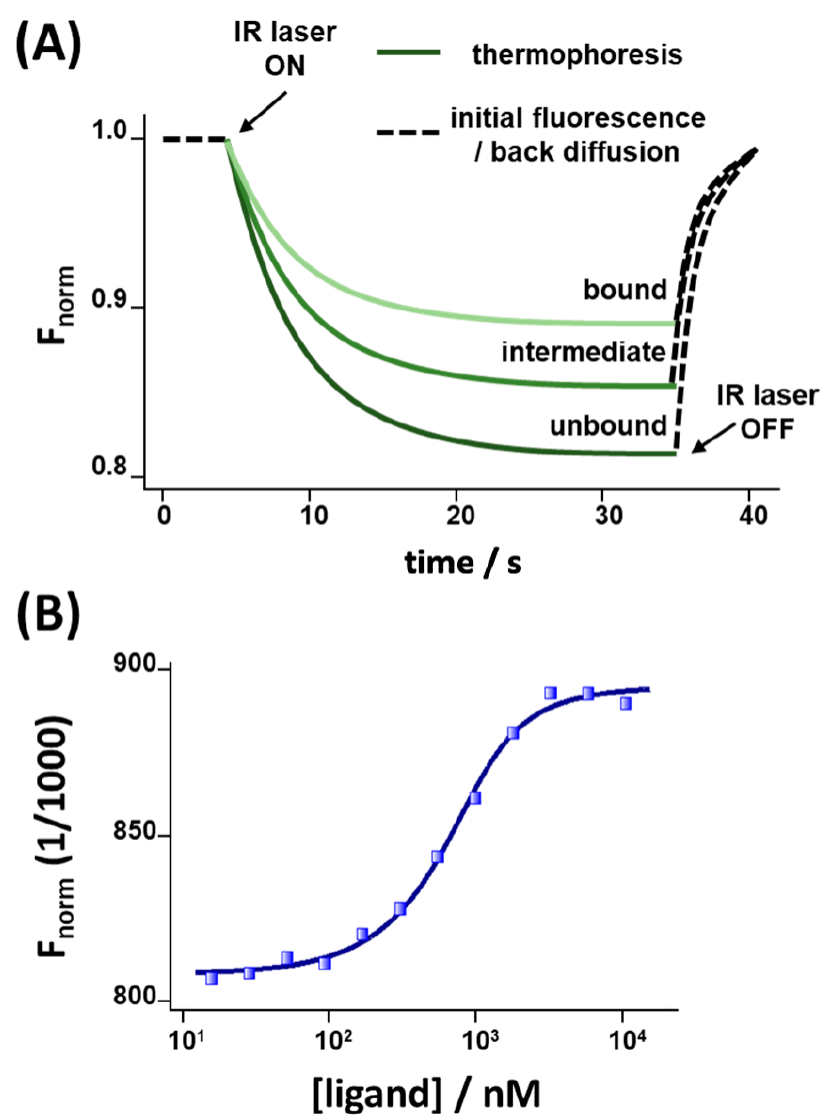
(A) Typical time-dependent MST curves associated with a fluorophore-modified receptor upon binding different concentrations of an affinity ligand. (B) Analysis of the experimental MST curves in terms of normalized fluorescence vs. the concentration of the ligand.

The computationally simulated structures and *K*_d_ values of the aptamer–peptide complexes were complemented by a quantitative evaluation of the binding affinity by microscale thermophoresis (MST) experiments, and the binding interactions and simulated structural features were further supported by NMR studies. The MST method receives broad recent analytical applications for probing the formation of supramolecular complexes and determining the dissociation constants of ligand–receptor complexes.^53,54^ The method relies on monitoring temporal fluorescence changes of a fluorophore-labeled probe on applying a gradient temperature change within a micrometer-sized volume spot. Typically, an IR laser induces a temperature change (ca. 4–5 °C) within the spot as compared to the bulk surrounding solution. This temperature difference induces the diffusion of the fluorescent probe through the solution (or the opposite diffusional process, depending on the Soret coefficient of the probe). The time-dependent fluorescence changes within the micrometer-sized probing spot are monitored by an analyzing beam. Typical temporal MST curves are depicted in Figure 1A. Upon switching on the heating laser, a time-dependent change in fluorescence intensity of the probe is observed due to the thermophoretic diffusion of the fluorescent label to the bulk that reaches a steady state when the thermophoretic force migrating the fluorescent label is counterbalanced by Brownian diffusion. Upon switching off the heating laser source, the original fluorescence within the probing spot is restored as the system re-equilibrates. The thermophoretic migration of the fluorescent probe is controlled by environmental effects (such as viscosity of the medium or ionic strength) and, most importantly, by the mass, shape, and solvation sphere of the diffusing probe, which are perturbed upon the supramolecular ligand/receptor complex formation, allowing the binding event of a small-molecule ligand to a receptor to be observed. From the thermophoretic fluorescence traces, the binding curves are derived by relating the normalized saturated fluorescence intensities to ligand concentration, Figure 1B. The dissociation constant (*K*_d_) of the resulting ligand–receptor complex is derived by fitting the experimental binding curve to eq 1, where *F* is the observed normalized fluorescence extracted from the MST curves, *K*_d_ is the dissociation constant, *c* is the ligand concentration, and *n* is the Hill coefficient, which takes into account any cooperative effects in the case of multiple ligand binding events.
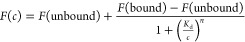
1

Indeed, MST provided a very useful tool for the evaluation of aptamer–ligand complexes.^55,56^ Specifically, for the present study, the DNA or RNA TAs were functionalized with the FAM fluorophore to allow us to make measurements by MST, and the MST curves were measured in the presence of a serial dilution of the peptide (or amino acid) ligands.

**Figure 2:**
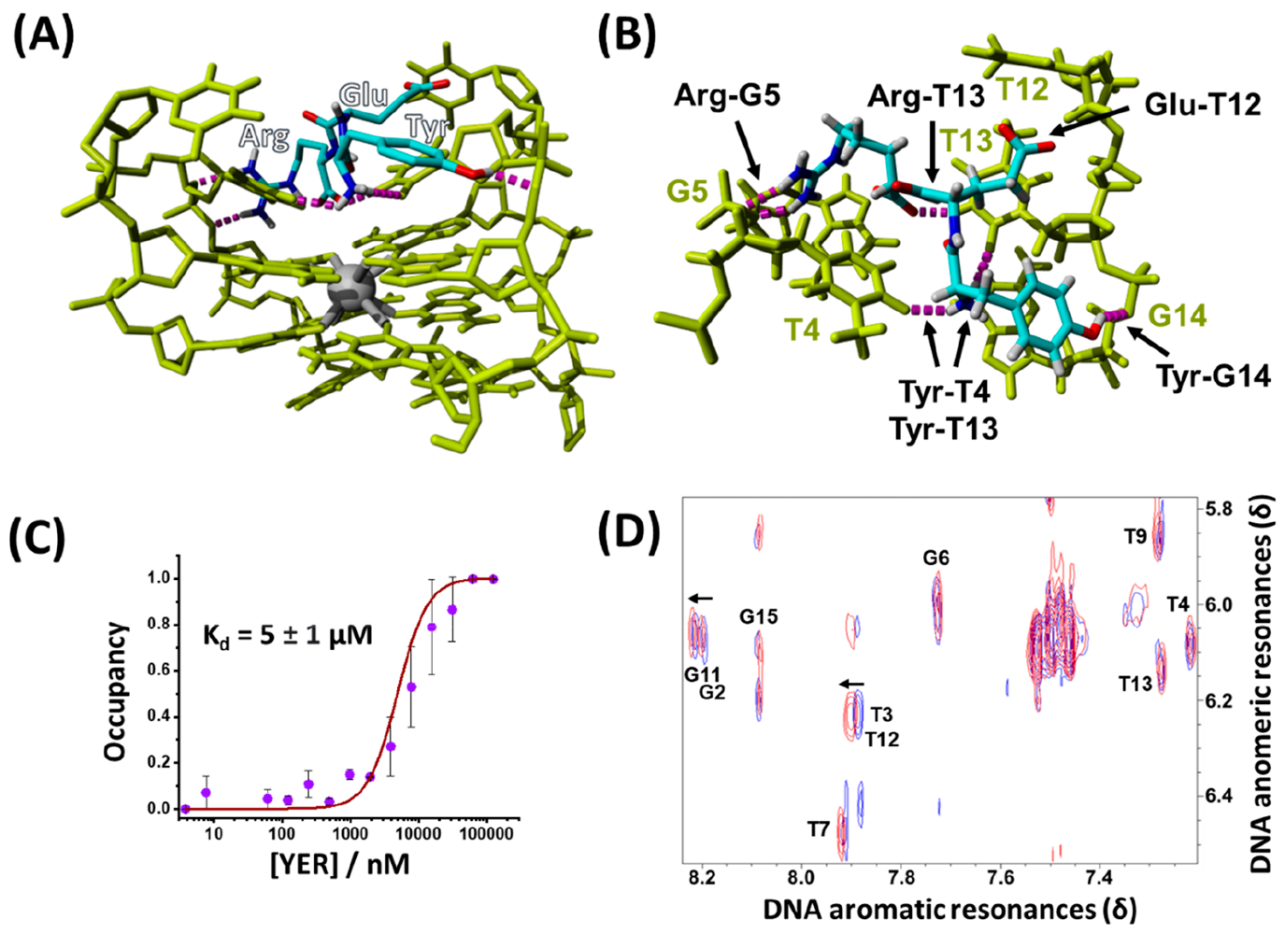
(A) Energy-minimized docked structure of the YER tripeptide to the DNA TA. (B) Enlarged top-down view of the binding pocket and H-bonding interactions between YER and specific bases associated with the DNA TA binding site. (C) Experimental MST binding curve corresponding to the association of YER to the DNA TA. (D) NOESY spectra showing aromatic/anomeric correlations of DNA TA in the absence of YER (blue) and after the formation of the YER/DNA TA affinity complex (red) upon adding 2 equivalents of YER. Labeling of resonances refers to aromatic protons assigned from published data.^49^

The three fragmented miniaturized tripeptides RYE, YER, and ERN were probed as potential ligands for the DNA TA in computational docking and MST experiments. As the most interesting results were obtained for the YER tripeptide, its interactions with the DNA TA will be addressed in detail in the body of the paper, while the interactions of the other two peptides will be presented in the Supporting Information and, where needed, will be compared to the YER binding properties. The energy-minimized simulated structure of the YER/DNA TA complex is displayed in Figure 2A. Figure 2B depicts the specific interactions observed between the DNA TA and the YER peptide. The tyrosine residue exhibits three hydrogen bonds to the aptamer residues: the phenolic OH forms a hydrogen bond with the phosphate backbone of G14 and its terminal NH_2_ residue with T4 and T13. The side-chain carboxyl group of the glutamic acid residue interacts with the pyrimidine base of T12. The terminal carboxyl moiety of the arginine residue shows a hydrogen-bonding interaction to the pyrimidine NH of the T13 base, while the guanidine moiety interacts with the T4 and G5 nucleotides. The YER binding site identified in the docking simulation, on the top face of the G-quadruplex within the T3/T4 and T12/T13 lateral loops, is similar to that observed in the native thrombin/DNA TA complex (Figure S1, Supporting Information). The computationally predicted dissociation constant is 12 μM. Figure 2C shows the MST-derived binding curve corresponding to YER/DNA TA complex formation. Representative MST traces are displayed in Figure S2 (Supporting Information). The derived dissociation constant of the complex corresponds to *K*_d_ = 5 ± 1 μM, in good agreement with that predicted by the simulations. The binding of YER to the aptamer reveals selectivity. Substitution of YER with structural analogues, such as FER and YEK, or further miniaturizing the tripeptide into YE or ER dipeptides, did not lead to the formation of peptide/DNA complexes. No binding curves could be detected in the respective MST experiments (Figure S3, Supporting Information).

Complementary ^1^H 1D and 2D NOESY NMR experiments further support the observed binding interaction and simulated structure of the YER/DNA TA complex. The solution structure of the thrombin aptamer determined by NMR methods was previously reported, allowing us to assign the DNA TA resonances.^49^Figure S4A (Supporting Information) provides a schematic structure of the DNA TA showing the 5′-3′ numbering of nucleotide residues. Comparison of the NOESY aromatic/anomeric correlations of the aptamer in the presence (2 equivalents) and absence of YER ligand, Figure 2D, reveals specific ligand-induced chemical shift perturbations of the G2, G11, T3, and T12 aromatic resonances corresponding to the top G-tetrad and two lateral loops that comprise the binding groove of the aptamer identified by the simulations, Figure S4B. Note that resonances corresponding to the lower face of the aptamer, for example, G6 and G15, are unperturbed indicating the ligand does not interact with this part of the structure. The aromatic/methyl correlations, Figure S4C, reveal specific chemical shift perturbations of the methyl resonances of T4 and T13, also belonging to the two lateral loops. Thus, it appears that the YER tripeptide occupies a similar aptamer binding site to the YER loop of the native protein (Figure S1). The imino resonances of the G-tetrads are also strongly perturbed by the addition of ligand, Figure S4D, providing further confirmation of the binding interaction of YER with the DNA TA.

The simulated energy-minimized structures of the ERN/DNA TA and RYE/DNA TA complexes, and the corresponding experimental MST binding curves and derived *K*_d_ values, are presented in Figure S5 (Supporting Information). ERN (*K*_d_ = 12 ± 1 μM) binds with a lower affinity to the DNA TA than YER, and the RYE tripeptide reveals a substantially lower binding affinity, *K*_d_ = 52 ± 5 μM, as compared to YER.

**Figure 3:**
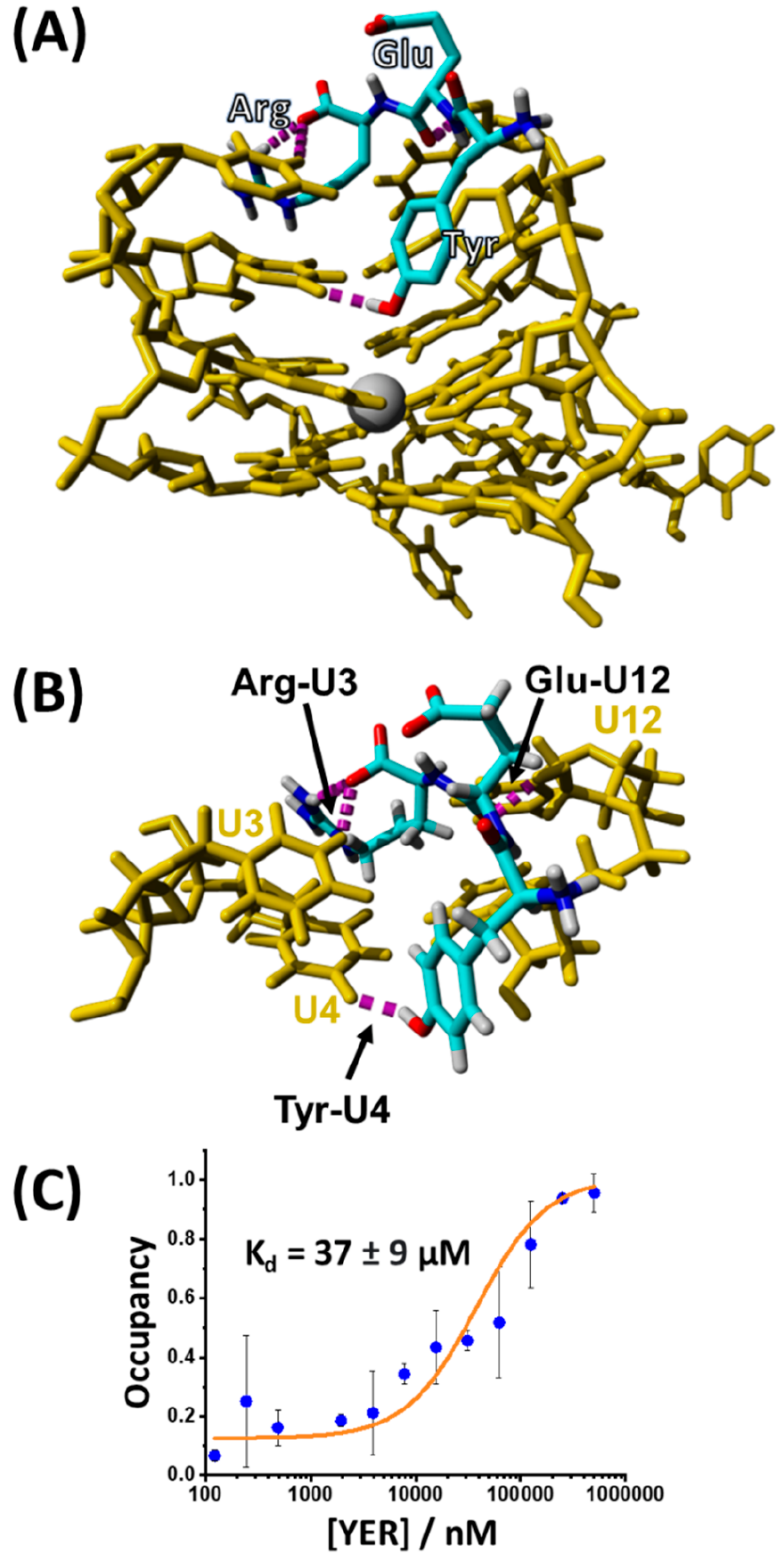
(A) Energy-minimized docked structure of the YER tripeptide to the RNA TA. (B) Enlarged top-down view of the binding pocket and H-bonding interactions between YER and specific bases associated with the RNA TA binding site. (C) Experimental MST binding curve corresponding to the association of YER to RNA TA.

In an attempt to understand the interactions between the miniaturized YER peptide with RNA aptamer sequences as a potential model to mimic RNA/peptide interaction under prebiotic conditions of an RNA/peptide world, we substituted DNA TA with analogous ribonucleotides to yield a potentially new RNA TA aptamer. Figure 3A shows the energy-minimized structure of the YER/RNA TA complex suggesting that the RNA sequence, indeed, may act as a binding aptamer for YER. In fact, the simulated structure suggests that the YER ligand occupies the same nucleotide pocket as in the case of DNA TA, although the specific interactions appear to be different, Figure 3B. For example, in this case the terminal carboxyl residue of arginine forms a hydrogen-bonding interaction with the pyrimidine NH moiety of U3, and the phenolic hydroxyl of tyrosine interacts with U4. Nonetheless, the interaction of YER with the groove formed by the two lateral loops above the upper G-tetrad is similar to that observed in the case of the DNA TA. Figure 3C shows the MST-derived binding curve of YER to the RNA TA. The derived dissociation constant corresponds to *K*_d_ = 37 ± 9 μM. That is, the binding affinity of YER to the RNA TA aptamer is lower than in the case of DNA TA.

**Figure 4:**
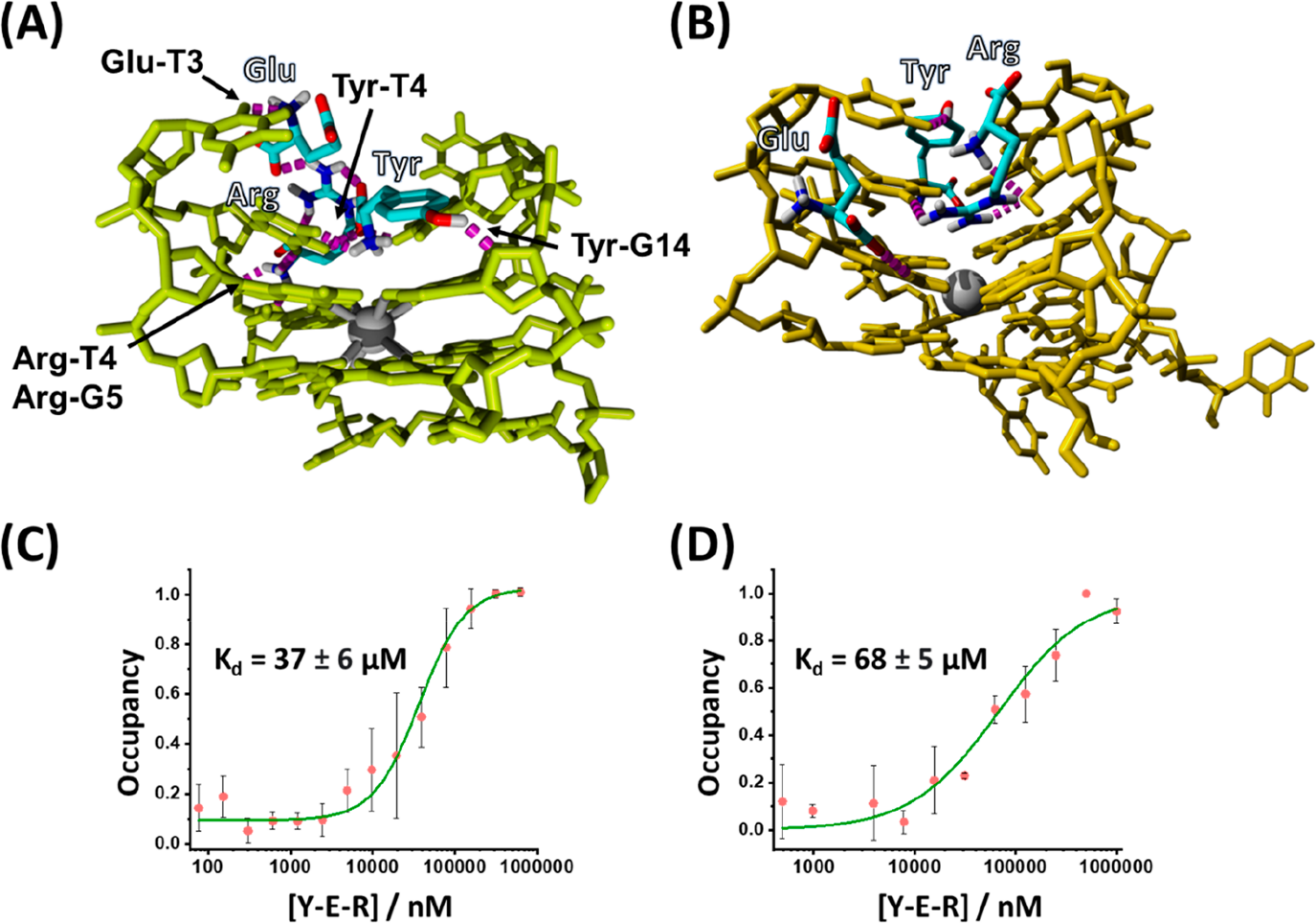
(A) Docked structure of the Y/E/R amino acids to the DNA TA. (B) Docked structure of the Y/E/R amino acids to the RNA TA. (C) Experimental MST binding curve corresponding to the association of YER to the DNA TA. (D) Experimental MST binding curve corresponding to the association of YER to the RNA TA.

Finally, we attempted to probe the possible interaction of the separate amino acids comprising the YER peptide with DNA TA and RNA TA. This experiment has significance, as it might shed light on the possible interactions of amino acids with DNA and RNA templates under prebiotic conditions and the possible evolution of peptides. Figure 4A,B shows the simulated structures of the Y/E/R clusters on the DNA TA and RNA TA, respectively. In the case of the DNA TA, Figure 4A, the three amino acids are found to form a viable cluster in the same DNA TA binding pocket as the YER tripeptide, following the same order and configuration. While the Tyr-G14, Tyr-T4, and Arg-G5 interactions are preserved, the glutamic acid residue is found to interact with T3 rather than T12, as observed for the tripeptide. Similarly, the structurally simulated docking experiment reveals that the three amino acids Y/E/R cluster in the RNA TA binding domain where the tripeptide YER binds, Figure 4B. The binding interactions between the amino acids seem to be, however, slightly different as compared to the clustering of the three amino acids to the DNA TA. While tyrosine and arginine occupy intimate spatial positions, the glutamic acid unit occupies a spatially separated position. Nonetheless, docking experiments of two separated amino acids Y/E, E/R, or Y/R to the DNA TA or RNA TA do not show stable clustering of the amino acids on the aptamers.

Following the docking simulations, we experimentally confirmed the results. Figure 4C,D depicts MST binding curves corresponding to the formation of three amino acid Y/E/R clusters to the DNA and RNA TAs. The dissociation constants correspond to 37 ± 6 and 68 ± 5 μM, respectively. While the dissociation constants are higher than the *K*_d_ values of the tripeptide YER/DNA TA and YER/RNA TA complexes, the results demonstrate the clustering phenomenon of the separated Y/E/R amino acids on the aptamers. The lower binding affinity of the three amino acid cluster with RNA TA, as compared to the binding of the cluster to the DNA TA, is consistent with the lower affinity of binding of the tripeptide YER to the RNA TA as compared to the binding of the tripeptide to the DNA TA.

The clustering effect is selective, and introduction of phenylalanine or lysine into the mixture or subjecting the DNA TA to only two (Y/E or E/R) amino acids did not reveal binding curves in the MST experiments. In addition, the single amino acids Y, E, and R lack binding affinity to the DNA TA (Figure S6, Supporting Information). Furthermore, we note that the separated amino acids R/Y/E or E/R/N, corresponding to the additional miniaturized tripeptides, do not cluster into stable complexes with the DNA/RNA TAs (confirmed by MST experiments).

As the DNA TA represents a chiral nucleic acid, the possible chiral discrimination of complex formation with d-tyrosine (D-Y), d-glutamic acid (D-E), and d-arginine (D-R) was addressed. Molecular docking simulations indicated that the three amino acids do not cluster on the DNA TA. In addition, MST measurements did not show any experimental binding curve, Figure S7 (Supporting Information), demonstrating chiral discrimination of the D-amino acid cluster formation with the DNA TA.

In order to support the generality of the concept where miniaturized peptides derived from a protein are identified as ligands revealing binging affinities toward the protein aptamer and, particularly, clustering as single amino acids with the antiprotein aptamer, we searched for other examples. Within these efforts we found that a domain of the multifunctional nucleolin protein (PDB ID: 2FC8)^57^ exhibits some structural similarities to thrombin. The structure contains a loop domain composed of a tripeptide, RET, where two amino acids (R, E) are common to the YER tripeptide composition that binds the thrombin DNA TA, while the third amino acid T contains an OH moiety in common with Y. Furthermore, like the thrombin binding aptamer, the AS1411 aptamer elicited against nucleolin exhibits a G-quadruplex structure.^58^ As the AS1411 aptamer is polymorphic^59^ we selected a modified version of the aptamer reported to form a single G-quadruplex topology (PDB ID: 2N3M)^42,43^ as the target aptamer for molecular docking and subsequent MST binding assays.

**Figure 5:**
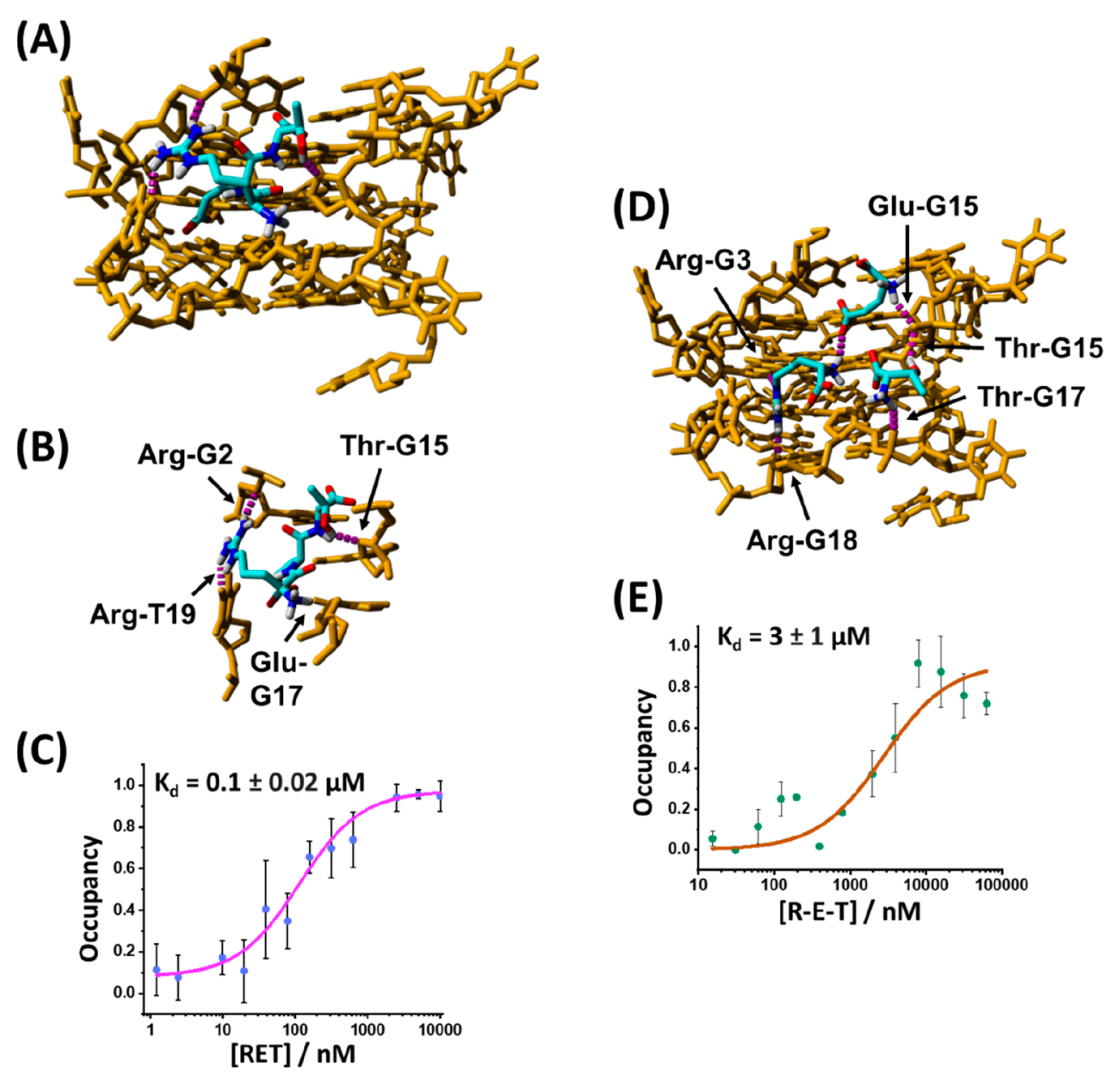
(A) Energy-minimized docked structure of the RET tripeptide to the 2N3M G-quadruplex. (B) Enlarged top-down view of the binding pocket and H-bonding interactions between RET and specific bases associated with the DNA binding site. (C) Experimental MST binding curve corresponding to the association of RET to the 2N3M G-quadruplex. (D) Docked structure and H-bonding interactions of the R/E/T amino acids to the 2N3M G-quadruplex. (E) Experimental MST binding curve corresponding to the association of R/E/T amino acids to the 2N3M G-quadruplex.

Figure 5A depicts the energy-minimized docked structure of the RET tripeptide against the 2N3M variant of the antinucleolin aptamer following 100 docking runs with the AutoDock VINA algorithm in the YASARA software. Interestingly, the peptide appears to target a side groove of the G-quadruplex rather than one of the terminal G-tetrads as observed in the case of YER to the DNA TA. Nonetheless, several interactions between the RET tripeptide and the aptamer are observed, Figure 5B. The guanidine moiety of the arginine residue forms H-bond interactions with G2 and T19. The glutamic acid residue interacts with G17. The secondary hydroxyl group of the threonine forms a H-bond to the G15 aptamer residue. Figure 5C shows the experimental MST-derived binding curve of RET to the 2N3M G-quadruplex. A dissociation constant of *K*_d_ = 0.1 ± 0.02 μM with the aptamer scaffold is detected. In this case, the experimental dissociation constant is significantly lower than that predicted by the docking simulation (*K*_d_ = 11 μM). The binding of RET to the 2N3M G-quadruplex aptamer is selective, and substitution of the RET to tripeptide compositions consisting of QET or REV reveals lack of affinity interactions of the mutated tripeptide to the 2N3M G-quadruplex aptamer (the Q substitutes the terminal guanidine moiety of R with an amide functionality, while the V mutation substitutes the hydroxyl of T with a methyl group), Figure S8, Supporting Information. The successful identification of the affinity complex between RET and 2N3M G-quadruplex aptamer challenged us to probe the possible clustering of the separated amino acids R/E/T at the G-quadruplex binding site. Figure 5D depicts the simulated clustered structure of the separated three amino acids R/E/T on the G-quadruplex binding domain. A viable three amino acid cluster in which the three amino acids occupy the same binding pocket as the RET tripeptide was observed, though slightly different interactions with the aptamer bases were seen. For example, the arginine residue contacts G3 and G18 as the single amino acid, rather than G2 and T19 in the case of the tripeptide. The interaction of threonine to G15 appears to be important in both structures. The identification of a potential clustered configuration of the R/E/T amino acids encouraged us to pursue MST experiments to validate the formation of an affinity cluster. Figure 5E shows the MST binding curve corresponding to the complex generated between the separated amino acids and the 2N3M G-quadruplex. The derived dissociation constant corresponds to *K*_d_ = 3 ± 1 μM. While the *K*_d_ value is 1 order of magnitude higher as compared to the *K*_d_ value of the RET tripeptide, it nonetheless demonstrates the formation of a supramolecular complex between the cluster of amino acids and the G-quadruplex. It should be noted that the single amino acids R, E, and T or combinations of the two amino acids R/E, E/T, or R/T do not show the formation of any affinity complexes with the G-quadruplex in the MST binding assay (Figure S9, Supporting Information), indicating the supramolecular interactions between the three amino acids play, indeed, a significant role in the clustering of single amino acids on the aptamer scaffold.

## Conclusions

The present study has introduced a method to utilize known aptamers as recognition strands for short peptide ligands and, eventually, strands that bind clusters of amino acids. The method involves the selection of a well-defined structure of an aptamer-protein complex and the identification of the peptide sequence in the aptamer responsible for the binding of the protein to the aptamer scaffold. The subsequent fragmentation of the peptide sequence followed by in silico docking-guided identification of the affinity complexes between the miniaturized peptides and the antiprotein aptamer, followed by experimental validation of the binding affinities between the fragmented peptide sequences and the antiprotein aptamer, leads to the identification of miniaturized peptides that bind to the parent protein aptamer. This method was exemplified by the identification of the pentapeptide RYERN as the functional scaffold that participates in the antithrombin DNA aptamer affinity complex. The pentapeptide structure was separated into three tripeptide fragments, RYE, YER, and ERN. In silico docking studies, followed by microscale thermophoresis (MST) binding experiments, demonstrated that the tripeptides RYE, YER, and ERN reveal, indeed, binding affinities toward the antithrombin aptamer, DNA TA. The binding properties were selective, and substitution of the tripeptides with foreign amino acids prohibited the formation of affinity complexes with the DNA TA. In addition, we demonstrated that the ribonucleotide-translated DNA TA into the RNA TA sequence yielded an active RNA aptamer that shows related binding properties toward the miniaturized tripeptide YER. Most importantly, and surprisingly, we demonstrated by the docking studies and experimental MST experiments that the separated amino acids Y/E/R assembled into a cluster that binds to the DNA TA aptamer. The docking experiments showed that the energy-minimized structure of the Y/E/R cluster follows the spatial configuration of the tripeptide YER on the DNA TA. Also, the cluster affinity complex is selective, and substitution of the amino acid mixture with foreign amino acids prevented the formation of affinity complexes with the DNA TA. This method to identify by in silico studies and MST experiments affinity complexes between miniaturized peptides or amino acid clusters and available antiprotein aptamers was further developed by the discovery that a derivative of the AS1411 antinucleolin aptamer acts as an active sequence that binds selectively the RET miniaturized tripeptide and induces the formation of a stable affinity complex with the cluster of separated amino acids R/E/T. The significance of the present study rests on the introduction of a potential method to apply known antiprotein aptamers as specific binding sequences for diverse miniaturized peptides and even selective clusters of amino acids. These results pave the way to further functionalize the miniaturized peptides with chemical constituents, such as redox-active, photoredox, or catalytic units, to yield diverse supramolecular aptamer–peptide structures. Furthermore, the study introduces new dimensions to structural affinity interactions between nucleic acids (DNA, RNA) and peptides or amino acids. In view of the broad interests of such nucleic acid/peptide/amino acid interactions in the evolution of life, the present study might provide tools to understanding the principles of prebiotic chemistry.

## Supporting Information Available

Structures of thrombin/aptamer complex, additional simulated docking poses, additional MST binding curves, MST control experiments, additional NMR analysis (PDF)
