# Supplementary material for: Aptamer–Protein Structures Guide In Silico and Experimental Discovery of Aptamer–Short Peptide Recognition Complexes or Aptamer–Amino Acid Cluster Complexes
Source: J Phys Chem B. 2022 Oct 31;126(44):8931–9. doi: 10.1021/acs.jpcb.2c05624 (PMC9661473; doi:10.1021/acs.jpcb.2c05624)
Supplement: Supplementary file 1 — jp2c05624_si_001.pdf [file jp2c05624_si_001.pdf]

SUPPORTING INFORMATION

Aptamer-Protein Structures Guide In Silico and Experimental Discovery of Aptamer - Short Peptide Recognition Complexes or Aptamer - Amino Acid Cluster Complexes

Michael Fadeev, Michael P. O'Hagan,

Yonatan Biniuri, and Itamar Willner*

The Institute of Chemistry, The Centre of Nanoscience and Nanotechnology

The Hebrew University of Jerusalem

Jerusalem 91904, Israel

* Corresponding author
itamar.willner@mail.huji.ac.il

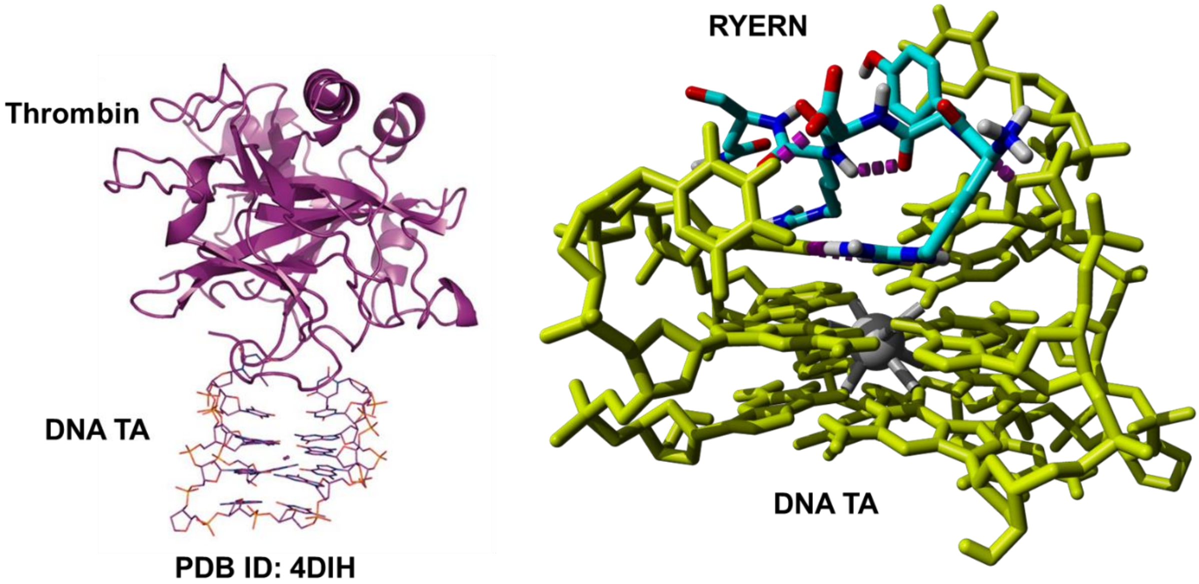

Figure S1: Reported structural features between the anti-thrombin DNA TA aptamer and its thrombin target. *Left*: The full crystallographic structure of the protein/aptamer complex (PDB: 4DIH).[1,2] *Right*: By inspection, we identified a key aptamer-recognition loop in the thrombin protein, composed of five amino acids (RYERN). The loop interacts primarily with the top face of the G-quadruplex, in particular, the two lateral loops (T3/T4, T12/13) forming several hydrogen bonds. A detailed analysis of the interactions was previously reported. Figure of 4DIH complex reprinted with permission, Copyright © 2012, Oxford University Press.[1]

(A)

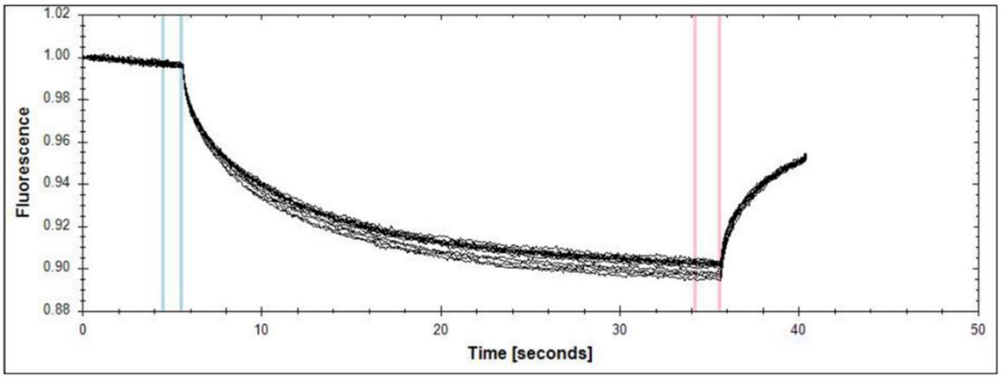

(B)

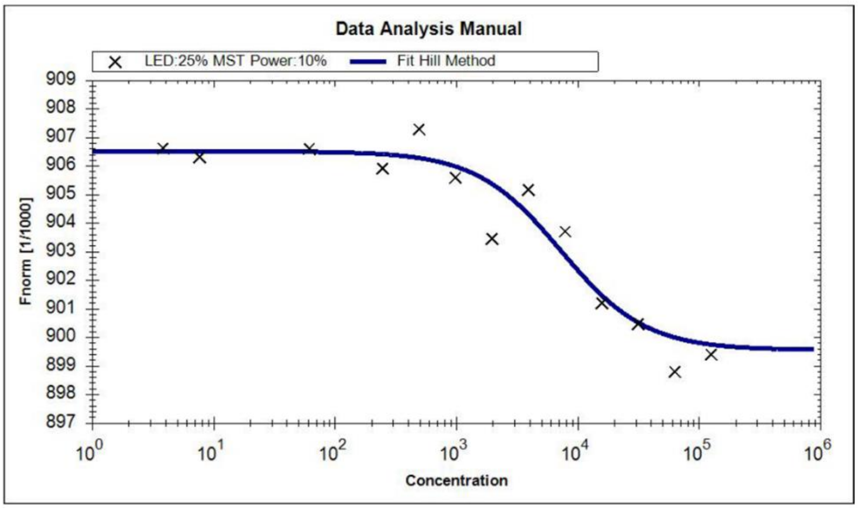

Figure S2: (A) Thermophoretic curves of FAM-DNA TA under varying YER tripeptide concentrations. (B), The normalized fluorescence values (F_{norm}) derived from thermophoretic curves as a function YER concentration (nM). X-axis is presented in log scale.

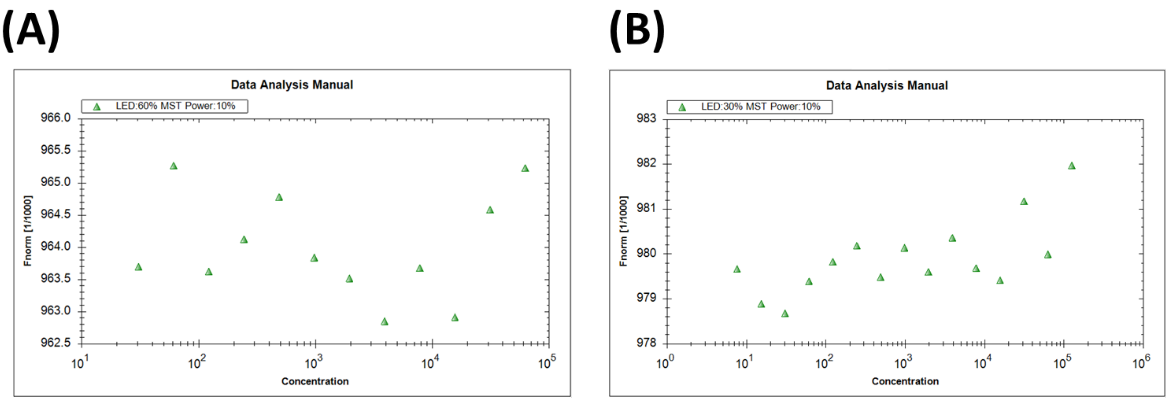

Figure S3: MST control experiments demonstrating absence of binding of (A) YE and (B) ER dipeptides to FAM-DNA TA. X-axis is dipeptide concentration (nM) presented in log scale.

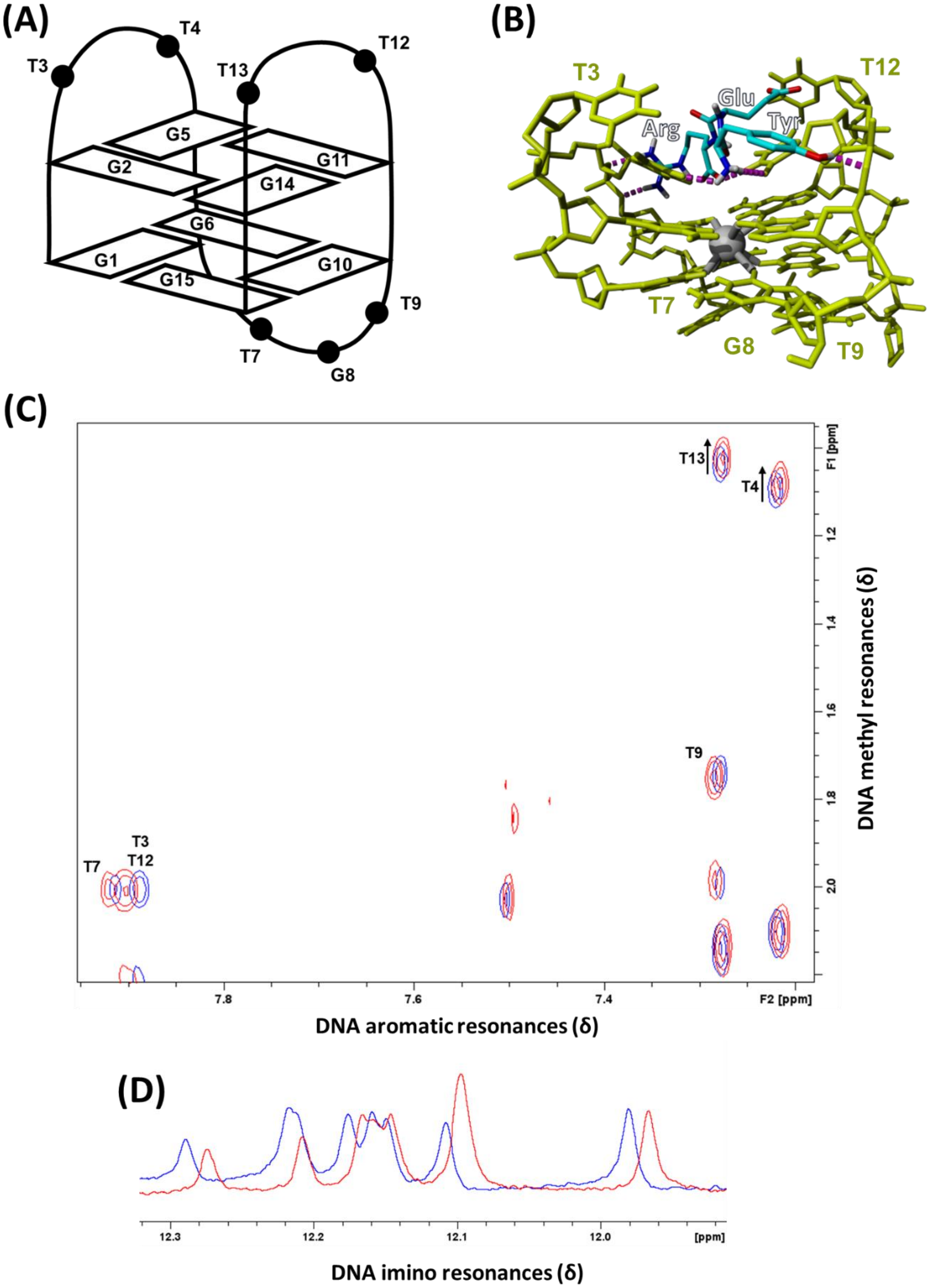

Figure S4: (A) Schematic representation of the DNA TA showing the 5'–3' numbered identification of the guanosine and thymidine bases creating the G-quadruplex structure. (B) Energy-minimized docked structure of YER associated with the DNA TA (side view). (C) ^{1}H

NOESY spectra showing aromatic/methyl correlations of the DNA TA in the absence of YER (blue) and after the formation of the YER/DNA TA affinity complex (red) upon adding 2 equiv. YER. Labelling of resonances refers to aromatic protons assigned from published data.[3] (D) 1D ^1H NMR spectra corresponding to the imino resonances of the DNA TA in the absence of YER (blue) and after the formation of the YER/DNA TA affinity complex (red) upon adding 2 equiv. YER.

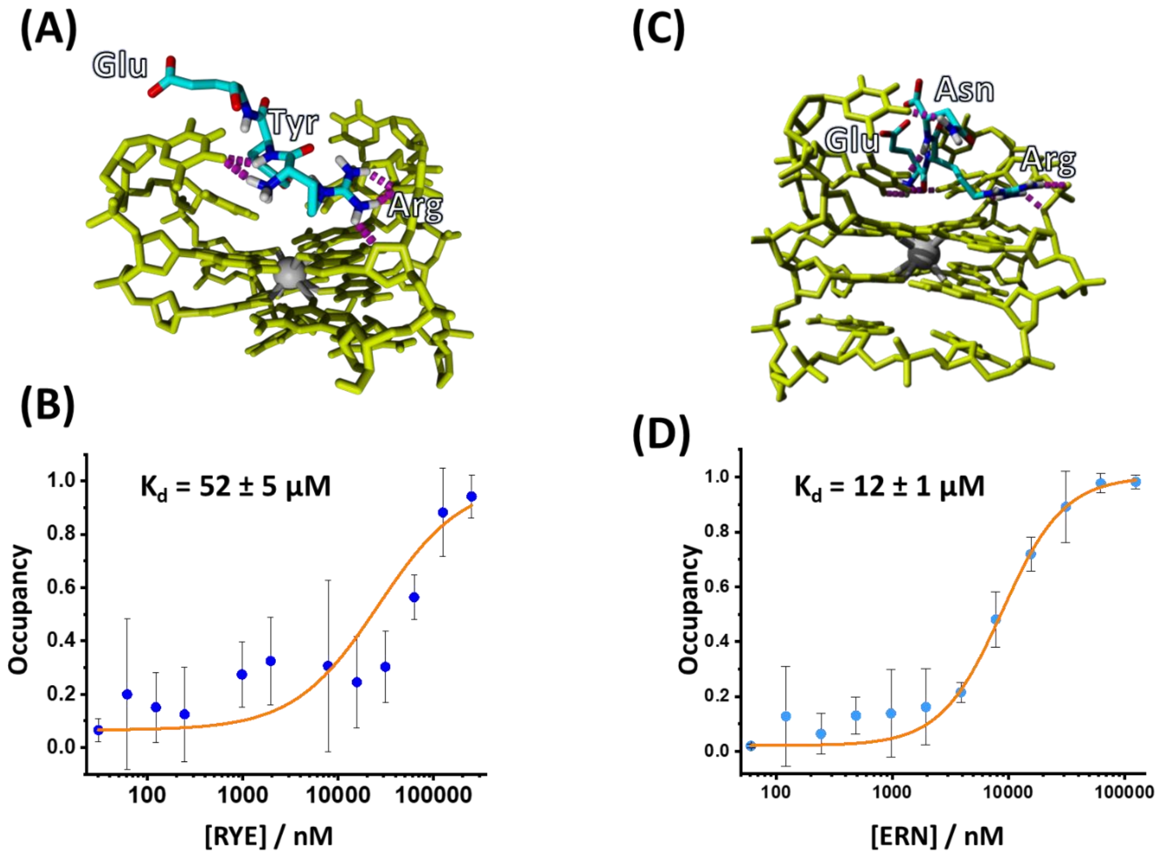

Figure S5: (A) Energy-minimized docked structure of the RYE tripeptide to the DNA TA. (B) Experimental MST binding curve corresponding to the association of RYE to the DNA TA. (C) Energy-minimized docked structure of the ERN tripeptide to the DNA TA. (D) Experimental MST binding curve corresponding to the association of ERN to the DNA TA.

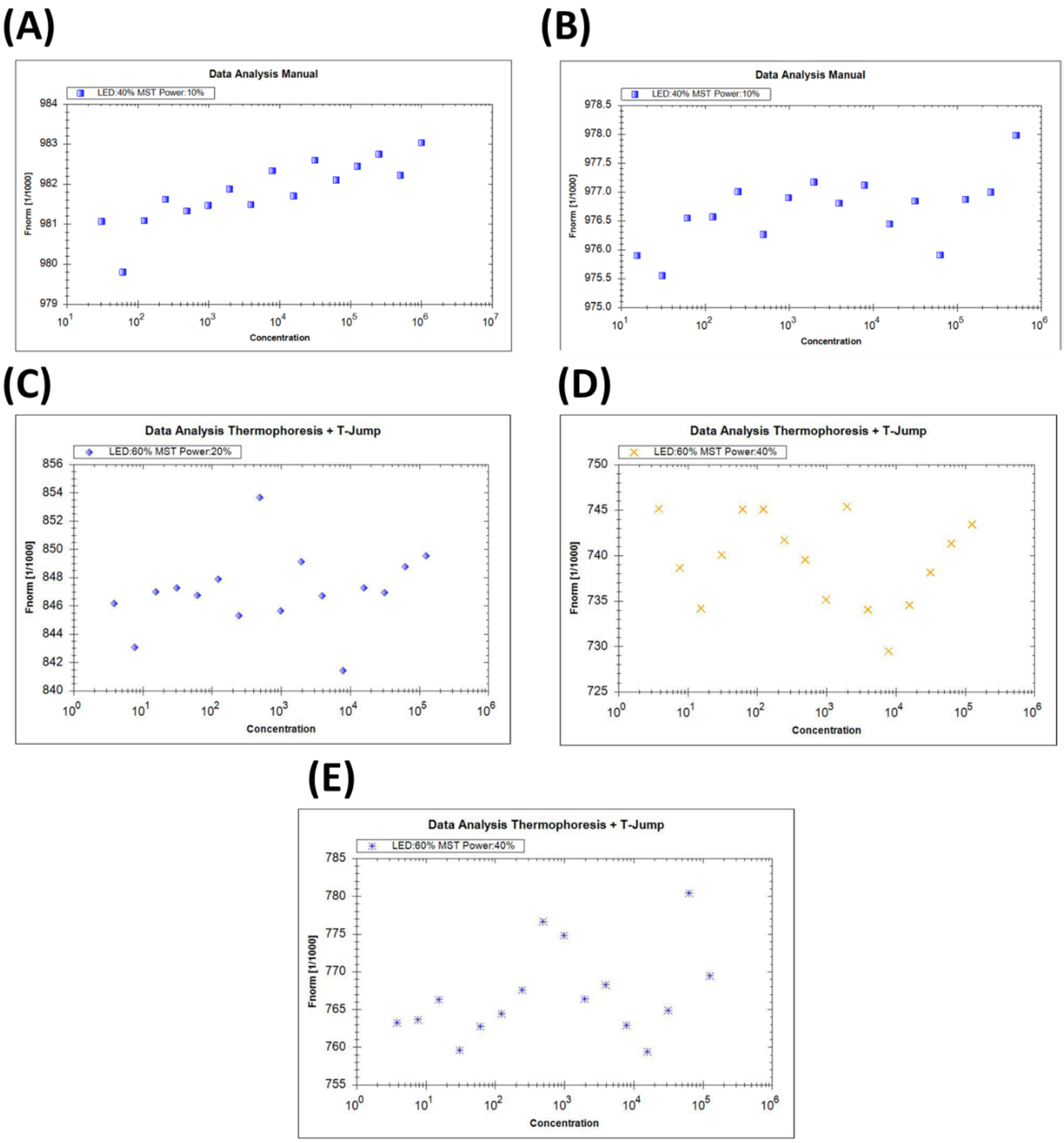

Figure S6: MST control experiments demonstrating absence of binding of (A) Y/E and (B) E/R amino acid mixtures and (C) Y, (D) E, (E) R single amino acids to FAM-DNA TA. X-axis is amino acid concentration (nM) presented in log scale.

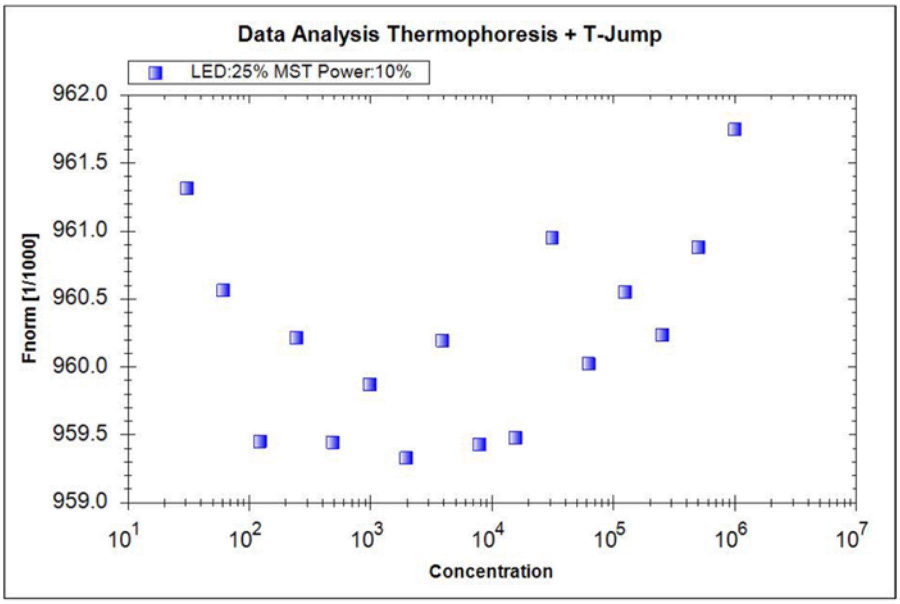

Figure S7: MST experiment demonstrating absence of binding of D-Y/D-E/D-R amino acid mixture to FAM-DNA TA. X-axis is amino acid concentration (nM) presented in log scale.

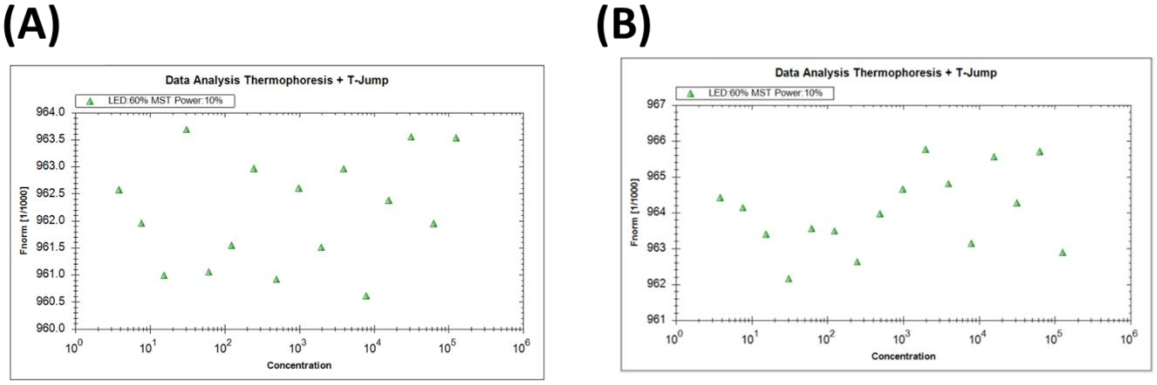

Figure S8: MST control experiments demonstrating absence of binding of (A) QET and (B) REV tripeptides to FAM-2N3M. X-axis is tripeptide concentration (nM) presented in log scale.

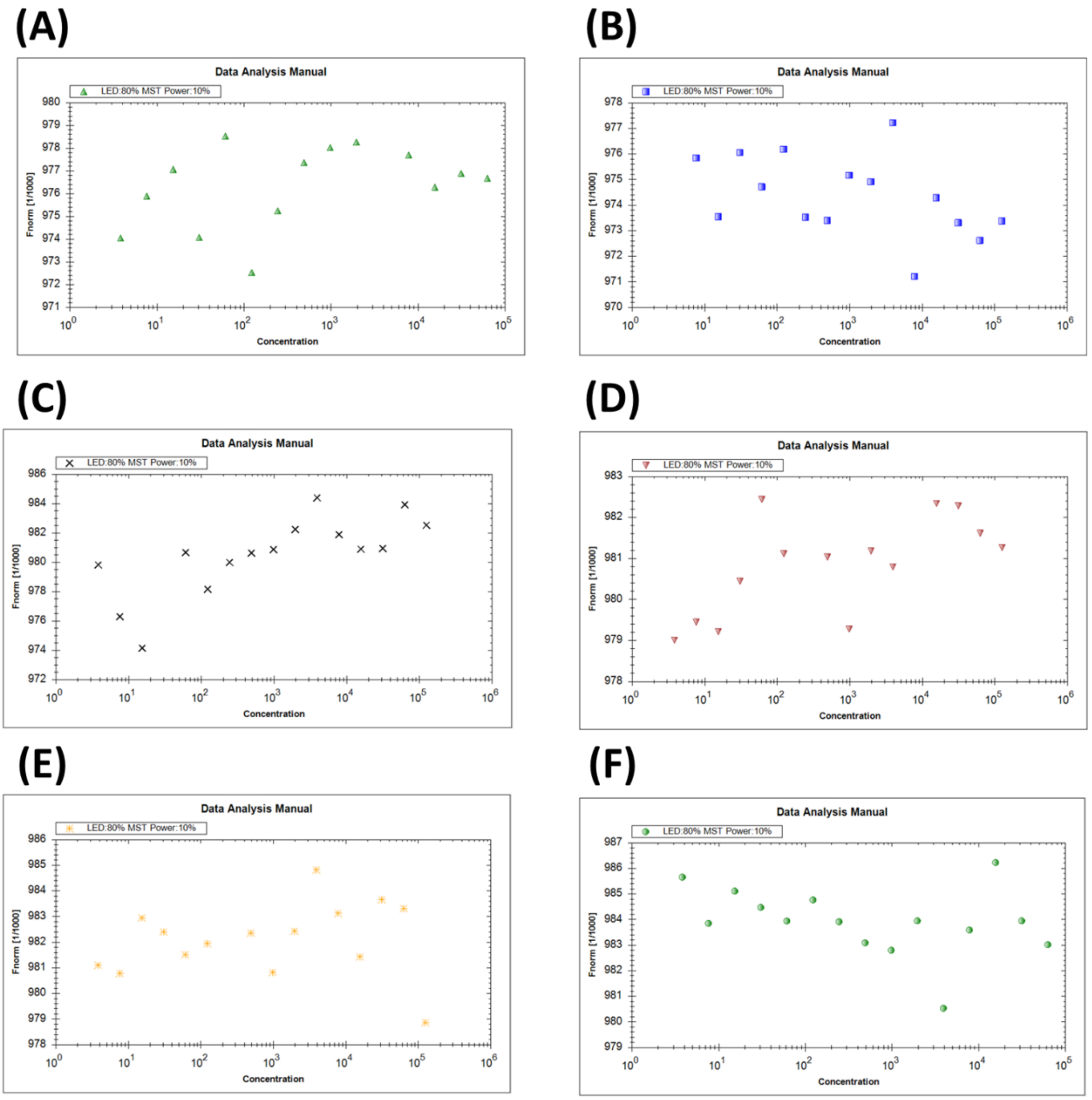

Figure S9: MST control experiments demonstrating absence of binding of (A) R, (B) E, (C) T single amino acids and (D) R+E, (E) E+T (F) R+T amino acid mixtures to FAM-2N3M. X-axis is amino acid concentration (nM) presented in log scale.

REFERENCES

(1) Russo Krauss, I.; Merlino, A.; Randazzo, A.; Novellino, E.; Mazzarella, L.; Sica, F. High-Resolution Structures of Two Complexes between Thrombin and Thrombin-Binding Aptamer Shed Light on the Role of Cations in the Aptamer Inhibitory Activity. *Nucleic Acids Res.* **2012**, *40* (16), 8119–8128.

(2) Russo Krauss, I.; Merlino, A.; Mazzarella, L.; Sica, F. X-Ray Structure of the Complex between Human Alpha Thrombin and Thrombin Binding Aptamer in the Presence of Sodium Ions. PDB DOI: 10.2210/Pdb4DIH/Pdb. **2012**.

(3) Schultze, P.; Macaya, R. F.; Feigon, J. Three-Dimensional Solution Structure of the Thrombin-Binding DNA Aptamer d(GGTTGGTGTGGTTGG). *J. Mol. Biol.* **1994**, *235* (5), 1532–1547.
